# Longitudinal clinical course, treatment outcomes, and relapse patterns in alopecia areata: a prospective cohort study

**DOI:** 10.3389/fmed.2025.1695618

**Published:** 2025-11-14

**Authors:** Yan Wenjie, Wang Yue, Wang Mengjiao, Zhao Yumei, Huang Xi

**Affiliations:** 1 Department of Dermatology and Venereology, The First Affiliated Hospital of Guilin Medical University, Guilin, China; 2 The First Affiliated Hospital of Guilin Medical University, Guilin, China

**Keywords:** alopecia areata, prospective cohort, Severity of Alopecia Tool (SALT), JAK inhibitors, quality of life

## Abstract

**Background:**

Alopecia areata (AA) is a common autoimmune hair loss disorder with a highly variable course and substantial psychosocial impact. Evidence from long-term prospective cohorts remains limited. To characterize the natural history, treatment outcomes, relapse patterns, quality-of-life changes, and safety profile of AA over 52 weeks in a real-world clinical setting.

**Methods:**

In this single-center prospective cohort, 400 patients with AA and 80 healthy controls were followed for 52 weeks, with assessments at weeks 4, 12, 24, 36, and 52. Disease severity was quantified using the Severity of Alopecia Tool (SALT). Primary outcomes included SALT50 and SALT90 achievement at week 24; secondary outcomes were relapse at week 52, patient-reported outcomes [Dermatology Life Quality Index (DLQI), Skindex-16, itch/pain numeric rating scale (NRS)], and safety. An imaging subcohort (*n* = 120) underwent dermoscopy and high-frequency ultrasound. Statistical analyses comprised mixed-effects models, logistic and Cox regression, ROC analyses, and correlation testing.

**Results:**

By week 52, 31.8% of patients relapsed, with a median relapse time of 28 weeks. SALT improved over time, with mean scores declining from 38.6 ± 21.7 at baseline to 22.4 ± 17.1 at week 52 (*P* < 0.001). At week 24, 62.5% achieved SALT50 and 28.3% achieved SALT90, with active disease, shorter duration, and younger age predicting better outcomes. ROC analysis showed improved discrimination for the enhanced model including activity and duration (AUC 0.81 vs. 0.69, *P* = 0.006). By week 52, 31.8% relapsed (median time 28 weeks), with relapse risk highest in those with prior episodes (HR 1.61, 95% CI 1.17–2.21). Quality-of-life indices improved significantly (DLQI −7.1, Skindex-16 −15.4 from baseline to week 52, both *P* < 0.001), and itch/pain reduction correlated with SALT improvement (*r* = 0.41 and 0.23, respectively). Dermoscopic and ultrasound parameters correlated with baseline severity and improved prediction of therapeutic response (AUC up to 0.84).

**Conclusion:**

This prospective cohort provides longitudinal evidence that baseline severity, disease activity, and relapse history are major determinants of response and recurrence in AA. Standardized severity scores, patient-reported outcomes, and imaging biomarkers may improve risk stratification, personalized care, and real-world evaluation of current therapies.

## Background

Alopecia areata (AA) is a common, non-scarring hair loss disorder characterized by the sudden onset of well-circumscribed, round or oval patches of alopecia (1–3). The disease course is highly variable, ranging from limited patchy hair loss to more extensive phenotypes such as alopecia totalis (AT) and alopecia universalis (AU) (4–6). Epidemiological studies have reported a global prevalence of approximately 0.1%–0.2%, with a lifetime risk approaching 2% (7–9). Although AA can occur at any age, it most frequently presents in adolescents and young adults, and it affects both sexes with nearly equal distribution (10). A growing body of evidence has also linked AA to an increased risk of psychiatric comorbidities, including anxiety and depression, underscoring its significant mental health impact (11–14). Consequently, AA is increasingly recognized not only as a dermatological disorder but also as a condition with broad psychosocial and public health implications (7, 10).

The pathogenesis of AA is widely regarded as an autoimmune process triggered by the collapse of hair follicle immune privilege (15, 16). In healthy conditions, hair follicles are protected from immune surveillance; however, disruption of this immune privilege exposes follicular antigens to immune recognition, leading to inflammatory attack (17–19). In parallel, T helper 1 (Th1) and Th17 pathways are strongly implicated, with cytokines such as interferon-γ (IFN-γ), and interleukin-17 (IL-17) serving as critical mediators of disease activity (20–22). Genome-wide association studies (GWAS) have identified multiple immune-related loci—including human leukocyte antigen (HLA) and cytotoxic T-lymphocyte–associated protein 4 (CTLA4)—that increase disease risk, supporting the hypothesis of immune dysregulation as a central mechanism (23, 24). Environmental triggers such as psychological stress, infections, and certain medications are further thought to precipitate or exacerbate disease onset in genetically predisposed individuals (25–27). Clinically, AA is highly heterogeneous, ranging from mild patchy hair loss to total scalp or body involvement (28). The course is often unpredictable, with alternating relapse and remission, and outcomes vary by disease duration, activity status, and relapse history (29).

Current management of AA relies primarily on corticosteroid-based therapies, including topical agents and intralesional triamcinolone, which remain the first-line treatments for limited disease (30, 31). In recent years, Janus kinase (JAK) inhibitors have shown promising efficacy in moderate-to-severe cases; however, questions remain regarding their long-term safety, durability of response, and relapse risk after discontinuation (32, 33). Despite therapeutic advances, important research gaps persist. There is a lack of prospective cohorts with long-term follow-up to systematically characterize the natural course of AA, recurrence patterns, and prognostic factors (34, 35). Moreover, relatively few studies have addressed patient-centered outcomes such as quality of life, symptom burden (itch and pain), or objective imaging biomarkers, leaving key aspects of disease impact and monitoring underexplored (36).

Given these gaps, a prospective cohort with standardized longitudinal assessments is essential to delineate the natural course of AA and to capture patterns of disease activity, remission, and relapse. By integrating clinical characteristics, quantitative imaging parameters, and patient-reported outcomes such as quality of life, such a study can also identify predictors of therapeutic response and relapse risk. Clinically, this approach has the potential to support risk stratification and individualized management strategies, while providing real-world evidence on the long-term safety and effectiveness of emerging treatments, including JAK inhibitors.

## Materials and methods

### Study design and setting

This was a prospective, single-center cohort study conducted in the Department of Dermatology at The First Affiliated Hospital of Guilin Medical University between January 2023 and December 2024. Participants were followed for a total of 52 weeks, with scheduled assessments at weeks 4, 12, 24, 36, and 52. The study protocol was approved by the institutional ethics committee, and all participants provided written informed consent prior to enrollment.

### Sample size estimation

The sample size was calculated based on the expected difference in the proportion of patients achieving SALT50 between subgroups, assuming response rates of 40% versus 25%. With a two-sided significance level of α = 0.05 and a statistical power of 80%, the estimated minimum sample size was 320 participants. To account for an anticipated dropout rate of approximately 20% during the 52-week follow-up, the final target enrollment was set at 400 patients.

### Inclusion criteria and exclusion criteria

Patients were eligible for enrollment if they met the following conditions: (1) a confirmed diagnosis of alopecia areata (AA) based on the international clinical guidelines proposed by Olsen et al.; (2) age between 12 and 65 years at the time of screening; and (3) willingness and ability to comply with study procedures, including scheduled visits and assessments, with provision of written informed consent (or assent with parental/guardian consent for minors).

Patients were excluded if they met any of the following conditions: (1) presence of other types of alopecia, including scarring alopecia, androgenetic alopecia, telogen effluvium, or traction alopecia; (2) concomitant severe systemic or dermatological conditions that could confound the assessment of AA or interfere with study participation, such as primary immunodeficiency, uncontrolled systemic infection, active malignancy, or significant hepatic or renal impairment; (3) history of systemic immunosuppressive therapy (e.g., corticosteroids, cyclosporine, methotrexate) or biologic agents (e.g., anti–TNF, anti–IL-17, JAK inhibitors) within the preceding 12 weeks; or (4) any other condition judged by the investigators to place the patient at unacceptable risk or to compromise the integrity of the study data.

### Grouping and subgroups

For exploratory analyses, patients were stratified into clinical subgroups according to baseline disease characteristics. Disease severity was categorized as mild or severe based on the extent of scalp involvement and corresponding SALT scores, following established clinical criteria. Disease activity was classified as active (progressive hair loss within the preceding 3 months) or stable (no evidence of recent progression). Duration of disease was dichotomized into ≤6 months versus >6 months from onset of first symptoms. In addition, patients were further classified as initial-onset or recurrent cases depending on whether they were experiencing their first episode or had a history of prior episodes of alopecia areata.

### Clinical and demographic information

At baseline, detailed demographic and clinical information was collected for all participants. Demographic variables included age, sex, and educational background. Clinical characteristics encompassed disease duration, age at onset, prior episodes of alopecia areata (relapse history), and family history of autoimmune or dermatological disorders. Data on previous and current treatments for AA, including topical, intralesional, systemic, or alternative therapies, were also systematically recorded. Relevant medical and dermatologic comorbidities were documented to assess potential confounders.

### Disease severity assessment

Disease severity was primarily assessed using the Severity of Alopecia Tool (SALT), which quantifies the percentage of scalp hair loss by dividing the scalp into four regions and assigning region-specific weights. SALT scores were obtained at baseline and at each scheduled follow-up visit (weeks 4, 12, 24, 36, and 52) to capture dynamic changes in disease extent over time. In addition, a predefined imaging subcohort underwent dermoscopic and high-frequency ultrasound evaluation. Dermoscopic parameters included hair density, terminal-to-vellus hair ratio, and the presence of black dots, which serve as indicators of follicular activity and disease severity. High-frequency ultrasound (20–50 MHz) was employed to assess dermal echogenicity, hair follicle morphology, and perifollicular blood flow signals, providing objective imaging biomarkers of disease activity.

### Therapeutic outcomes

The primary therapeutic outcomes were the proportions of patients achieving at least 50% improvement (SALT50) and 90% improvement (SALT90) in scalp hair regrowth relative to baseline, assessed at week 24. These thresholds are widely used as clinically meaningful indicators of treatment response in AA clinical trials. The secondary outcomes included the relapse rate at week 52 and the time to relapse, defined as the recurrence of ≥25% hair loss after achieving SALT50. Relapse-free survival was estimated using the Kaplan–Meier method, with subgroup comparisons performed by log-rank tests.

### Quality of life and symptom assessments

Health-related quality of life was evaluated using the Dermatology Life Quality Index (DLQI) and the Skindex-16, both of which are validated instruments for dermatological conditions. The DLQI comprises 10 items covering symptoms, daily activities, leisure, work/school, personal relationships, and treatment-related burden, with higher scores reflecting greater impairment. The Skindex-16 further assesses symptoms, emotions, and functional limitations on a broader scale. In addition, patient-reported symptom burden was assessed using a Numeric Rating Scale (NRS) for pruritus and pain, ranging from 0 (“no symptom”) to 10 (“worst imaginable symptom”). These instruments were administered at baseline and at each scheduled follow-up visit to capture temporal changes and their correlation with clinical severity.

### Safety assessments

Safety outcomes were systematically monitored throughout the study. All adverse events (AEs) were recorded, with specific attention to local corticosteroid-related reactions (such as skin atrophy, telangiectasia, or folliculitis) and systemic adverse effects associated with JAK inhibitors (including infection, gastrointestinal disturbances, or hematologic abnormalities).

### Statistical analysis

All statistical analyses were performed using SPSS and R. Continuous variables were summarized as means ± standard deviations (SD) for normally distributed data, or as medians with interquartile ranges (IQRs) for skewed data. Categorical variables were presented as frequencies and percentages. Between-group comparisons were conducted using the independent-samples *t*-test or Mann–Whitney *U* test for continuous variables, and the χ^2^ test for categorical variables, as appropriate.

Longitudinal changes in disease severity (SALT scores) were analyzed using linear mixed-effects models (LMM), which can accommodate missing-at-random observations without requiring imputation. For other analyses (e.g., logistic regression at week 24), only participants with complete outcome data at the respective time point were included; no last observation carried forward (LOCF) or other imputation methods were applied. Therapeutic outcomes were evaluated by first performing univariate analyses (χ^2^ test or *t*-test), followed by multivariable logistic regression models to identify independent predictors of achieving SALT50 and SALT90 at week 24, with results expressed as odds ratios (ORs) and 95% confidence intervals (CIs). The discriminative ability of baseline versus enhanced predictive models was assessed using receiver operating characteristic (ROC) curves, with area under the curve (AUC) values compared by the DeLong test.

Relapse outcomes were assessed using Kaplan–Meier survival analysis to estimate relapse-free survival, with subgroup comparisons performed using the log-rank test. Cox proportional hazards regression models were used to identify independent predictors of relapse, with hazard ratios (HRs) and 95% CIs reported. Quality of life and symptom outcomes were analyzed using paired *t*-tests or Wilcoxon signed-rank tests to assess within-group changes. Associations between changes in numeric rating scale (NRS) scores for itch and pain and changes in SALT scores were evaluated using Spearman correlation coefficients. Safety outcomes were summarized descriptively as incidence rates of adverse events, with subgroup analyses stratified by treatment modality, age, and sex. A two-sided *P*-value < 0.05 was considered statistically significant for all analyses.

## Results

### Study population and baseline characteristics

A total of 400 patients with alopecia areata (AA) and 80 age- and sex-matched healthy controls were included in the analysis. The mean age of the AA cohort was 32.74 ± 9.58 years, which was comparable to the control group (33.41 ± 8.86 years, *P* = 0.573). The proportion of female participants was also similar between groups (55.3% vs. 52.5%, *P* = 0.684). Mean body mass index (BMI) did not differ significantly between patients and controls (23.87 ± 3.27 vs. 23.46 ± 3.05 kg/m^2^, *P* = 0.412). Detailed demographic and clinical characteristics are summarized in Table 1.

**TABLE 1:** Baseline characteristics of patients with alopecia areata and healthy controls.

Characteristic	AA cohort (*n* = 400)	Healthy controls (*n* = 80)	*P*-value
Age, years (mean ± SD)	32.74 ± 9.58	33.41 ± 8.86	0.573
Female sex, *n* (%)	221 (55.3)	42 (52.5)	0.684
BMI, kg/m^2^ (mean ± SD)	23.87 ± 3.27	23.46 ± 3.05	0.412
**Disease duration**			
≤ 6 months, *n* (%)	212 (53.0)	–	–
> 6 months, *n* (%)	188 (47.0)	–	–
**Disease activity**			
Active, *n* (%)	219 (54.8)	–	–
Stable, *n* (%)	181 (45.2)	–	–
**Severity (SALT categories)**			
Mild (SALT ≤ 25%)	139 (34.8)	–	–
Moderate (26%–49%)	91 (22.8)	–	–
Severe (≥ 50%)	170 (42.5)	–	–
History of relapse, *n* (%)	118 (29.5)	–	–
**Comorbidities, *n* (%)**			
Atopic dermatitis	37 (9.3)	3 (3.8)	0.048*
Thyroid disorders	29 (7.3)	4 (5.0)	0.356
Anxiety/depression	62 (15.5)	7 (8.8)	0.071
Baseline DLQI score (mean ± SD)	11.27 ± 5.62	2.91 ± 1.84	<0.001**

Values are mean ± SD or *n* (%). SALT, Severity of Alopecia Tool; DLQI, Dermatology Life Quality Index.

*P-values from independent *t*-test or χ^2^ test as appropriate. *p < 0.05, *p < 0.01,

**p < 0.001.

With regard to disease characteristics, 53.0% of patients presented with a disease duration of ≤6 months, whereas 47.0% had a duration >6 months. More than half of the patients (54.8%) were classified as having active disease at enrollment, while 45.2% were in a stable phase. Based on SALT categories, 34.8% of patients had mild disease (≤25% scalp involvement), 22.8% had moderate disease (26%–49%), and 42.5% had severe disease (≥50%). A history of relapse was reported in 29.5% of cases.

Regarding comorbid conditions, atopic dermatitis was more frequent in patients with AA compared to controls (9.3% vs. 3.8%, *P* = 0.048). Thyroid disorders (7.3% vs. 5.0%, *P* = 0.356) and anxiety or depression (15.5% vs. 8.8%, *P* = 0.071) were more prevalent in the AA group but did not reach statistical significance. Importantly, the baseline Dermatology Life Quality Index (DLQI) score was markedly higher in patients with AA than in controls (11.27 ± 5.62 vs. 2.91 ± 1.84, *P* < 0.001), indicating a substantial impairment of health-related quality of life associated with the disease.

### Baseline SALT scores across clinical subgroups

At baseline, the mean SALT score in the overall cohort was 38.6 ± 21.7. Patients with a disease duration >6 months showed slightly higher SALT scores compared to those with a shorter duration (40.21 ± 22.05 vs. 37.12 ± 21.34), although this difference was not statistically significant (*P* = 0.142). By contrast, patients in the active phase had substantially higher SALT scores than those in the stable phase (45.83 ± 20.94 vs. 30.42 ± 19.18, *P* < 0.001). Likewise, patients with a history of relapse exhibited significantly greater severity compared with those without relapse (42.87 ± 22.31 vs. 36.02 ± 20.79, *P* = 0.018) (Table 2 and Supplementary Figure 1).

**TABLE 2:** Baseline SALT scores across clinical subgroups of alopecia areata.

Subgroup	*n*	SALT score, mean ± SD	*P*-value
**Disease duration**			
≤ 6 months	212	37.1 ± 21.3	0.142
> 6 months	188	40.2 ± 22.1	
**Disease activity**			
Active	219	45.8 ± 20.9	<0.001**
Stable	181	30.4 ± 19.2	
**History of relapse**			
Yes	118	42.9 ± 22.3	0.018*
No	282	36.0 ± 20.8	

Values are mean ± SD. SALT, Severity of Alopecia Tool.

*P-values were calculated using independent *t*-tests. *p < 0.05, *p < 0.01,

**p < 0.001.

### Longitudinal changes in SALT scores

**FIGURE 1:**
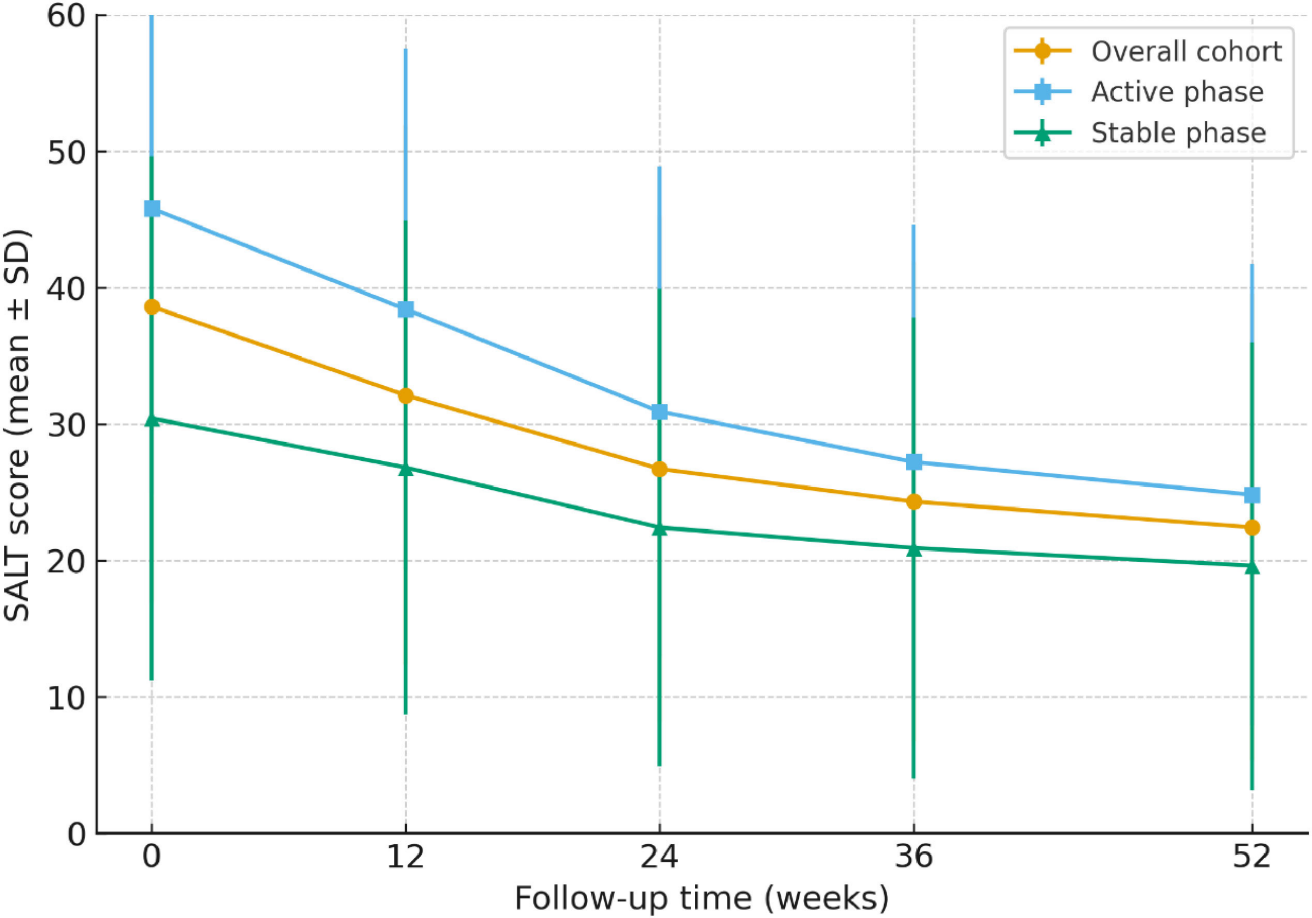
Longitudinal trajectories of SALT scores over 52 weeks in patients with alopecia areata. Mean SALT scores (±SD) are shown for the overall cohort (*n* = 400), the active subgroup (*n* = 219), and the stable subgroup (*n* = 181) at baseline, and at weeks 12, 24, 36, and 52. Patients in the active phase demonstrated higher baseline severity and a steeper decline in SALT scores over time compared with those in the stable phase. Mixed-effects modeling confirmed significant time × activity interactions (*P* = 0.004), indicating that baseline disease activity modified the longitudinal course of severity.

During the 52-week follow-up, SALT scores declined progressively in the overall cohort, with mean values decreasing from 38.6 ± 21.7 at baseline to 32.1 ± 19.8 at week 12, 26.7 ± 18.5 at week 24, 24.3 ± 17.6 at week 36, and 22.4 ± 17.1 at week 52 (*P* for trend < 0.001). The trajectories of change are shown in Figure 1. When stratified by disease activity, patients in the active phase exhibited a greater reduction in SALT scores compared with those in the stable phase. Similarly, patients with shorter disease duration (≤6 months) showed a steeper decline than those with longer disease duration (>6 months). Mixed-effects model analysis confirmed significant time × activity (*P* = 0.004) and time × duration (*P* = 0.031) interactions, indicating that both baseline activity status and disease duration modified the longitudinal course of severity.

### Response rates across subgroups

**FIGURE 2:**
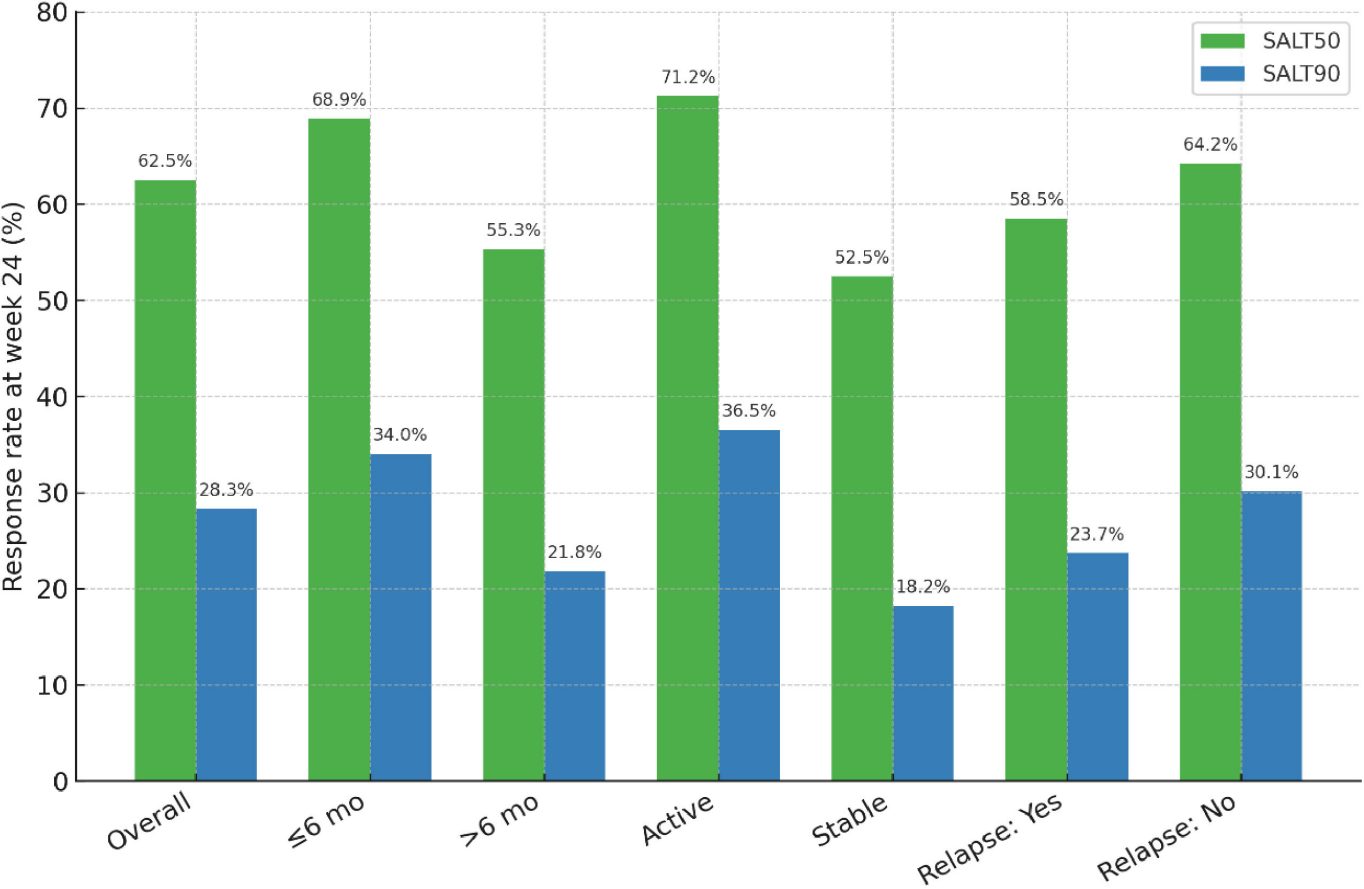
Response rates at week 24 across clinical subgroups of alopecia areata. Proportion of patients achieving SALT50 (≥50% improvement from baseline) and SALT90 (≥90% improvement) at week 24. Overall, 62.5% of patients reached SALT50 and 28.3% reached SALT90.

At week 24, 62.5% (250/400) of patients achieved SALT50 and 28.3% (113/400) achieved SALT90. These overall response rates are summarized in Table 3. When stratified by disease duration, patients with ≤6 months showed significantly higher SALT50 and SALT90 attainment than those with longer disease duration (68.9% vs. 55.3%, *P* = 0.018; 34.0% vs. 21.8%, *P* = 0.041). With respect to disease activity, patients in the active phase had markedly superior response rates compared with those in the stable phase (SALT50: 71.2% vs. 52.5%, *P* = 0.002; SALT90: 36.5% vs. 18.2%, *P* < 0.001). A history of relapse was associated with lower—but non-significant—response rates (SALT50: 58.5% vs. 64.2%, *P* = 0.281; SALT90: 23.7% vs. 30.1%, *P* = 0.164). The distribution of treatment responses is further illustrated in Figure 2.

**TABLE 3:** SALT50 and SALT90 response rates at week 24 across clinical subgroups.

Subgroup	*n*	SALT50, n (%)	SALT90, n (%)	*P*-value (SALT50)	*P*-value (SALT90)
Overall cohort	400	250 (62.5)	113 (28.3)	–	–
**Disease duration**					
≤6 months	212	146 (68.9)	72 (34.0)	0.018*	0.041*
>6 months	188	104 (55.3)	41 (21.8)		
**Disease activity**					
Active	219	156 (71.2)	80 (36.5)	0.002**	<0.001**
Stable	181	95 (52.5)	33 (18.2)		
**History of relapse**					
Yes	118	69 (58.5)	28 (23.7)	0.281	0.164
No	282	181 (64.2)	85 (30.1)		

*Values are *n* (%). SALT50, ≥50% improvement in SALT score; SALT90, ≥90% improvement. *P*-values from χ^2^ test. *p < 0.05, *p < 0.01,

**p < 0.001.

### Univariate analysis of predictors

Univariate testing identified several predictors of SALT50 achievement at 24 weeks (Table 4). Responders were significantly younger than non-responders (31.42 ± 9.15 vs. 34.91 ± 10.02 years, *P* = 0.007). Shorter disease duration (≤6 months, *P* = 0.018), active disease (*P* < 0.001), and absence of atopic dermatitis (*P* = 0.004) were also associated with higher response rates. In contrast, sex, BMI, history of relapse, thyroid disorder, and anxiety/depression showed no significant associations.

**TABLE 4:** Univariate analysis of baseline factors associated with SALT50 achievement at week 24.

Variable	SALT50 achieved (*n* = 250)	No SALT50 (*n* = 150)	*P*-value
Age, years (mean ± SD)	31.42 ± 9.15	34.91 ± 10.02	0.007**
Sex, female, *n* (%)	142 (56.8)	79 (52.7)	0.441
BMI, kg/m^2^ (mean ± SD)	23.61 ± 3.12	24.19 ± 3.36	0.116
**Disease duration**			
≤ 6 months, *n* (%)	146 (58.4)	66 (44.0)	0.018*
> 6 months, *n* (%)	104 (41.6)	84 (56.0)	
**Disease activity**			
Active, *n* (%)	156 (62.4)	63 (42.0)	<0.001**
Stable, *n* (%)	94 (37.6)	87 (58.0)	
**History of relapse**			
Yes, *n* (%)	69 (27.6)	49 (32.7)	0.281
No, *n* (%)	181 (72.4)	101 (67.3)	
Atopic dermatitis, *n* (%)	14 (5.6)	23 (15.3)	0.004**
Thyroid disorder, *n* (%)	18 (7.2)	11 (7.3)	0.972
Anxiety/depression, *n* (%)	33 (13.2)	29 (19.3)	0.122

*Values are mean ± SD or *n* (%). *P*-values derived from independent *t*-test or χ^2^ test as appropriate. *p < 0.05, *p < 0.01,

**p < 0.001.

### Multivariable logistic regression

Multivariate logistic regression (Table 5) confirmed that active disease (OR 2.14, 95% CI 1.46–3.14, *P* < 0.001) and shorter disease duration (OR 1.62, 95% CI 1.11–2.36, *P* = 0.013) were independent predictors of treatment response. Younger age also remained a significant factor (OR 0.97 per year, 95% CI 0.95–0.99, *P* = 0.011), whereas the presence of atopic dermatitis predicted lower odds of response (OR 0.48, 95% CI 0.24–0.93, *P* = 0.029).

**TABLE 5:** Multivariate logistic regression analysis of predictors of SALT50 achievement at week 24.

Variable	OR	95% CI	*P*-value
Age, years	0.97	0.95–0.99	0.011*
Disease duration ≤ 6 mo	1.62	1.11–2.36	0.013*
Active disease	2.14	1.46–3.14	<0.001**
Atopic dermatitis	0.48	0.24–0.93	0.029*

*OR, odds ratio; CI, confidence interval. Logistic regression model adjusted for age, disease duration, disease activity, and atopic dermatitis. *p < 0.05, *p < 0.01,

**p < 0.001.

### Model discrimination and ROC analysis

The baseline prediction model, including only demographic and clinical covariates, showed modest discrimination with an AUC of 0.69 (95% CI: 0.64–0.74). By contrast, the enhanced model, which additionally incorporated disease activity and disease duration, achieved an AUC of 0.81 (95% CI: 0.76–0.85). The improvement was statistically significant by DeLong’s test (*P* = 0.006).

**FIGURE 3:**
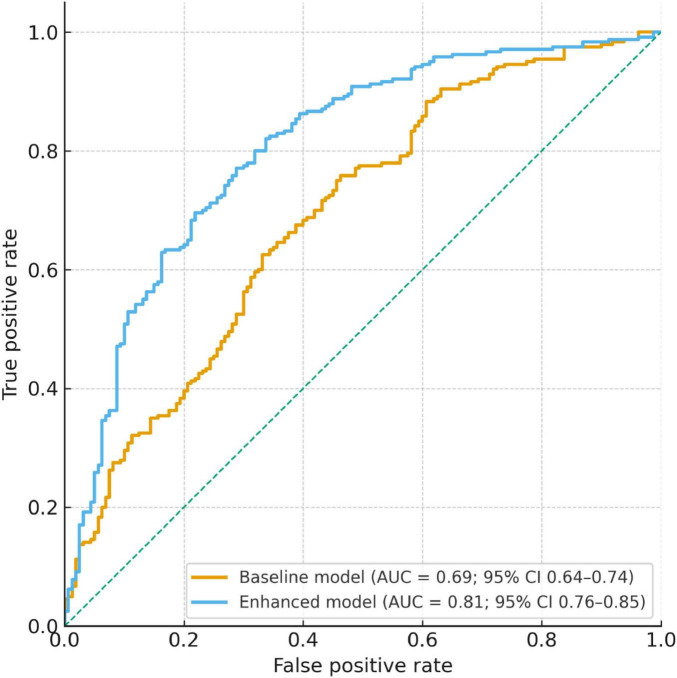
ROC curve analysis of baseline and enhanced models for predicting SALT50 achievement at week 24. Receiver operating characteristic (ROC) curves comparing the baseline model and the enhanced model. The baseline model, including demographic and clinical covariates, yielded an AUC of 0.69 (95% CI: 0.64–0.74). The enhanced model, which additionally incorporated disease activity and disease duration, demonstrated superior discrimination with an AUC of 0.81 (95% CI: 0.76–0.85). The enhanced model significantly outperformed the baseline model (DeLong test, *P* = 0.006).

Operating characteristics of the two models are summarized in Table 6. At the optimal cut-off (Youden index), sensitivity and specificity were 71.2% and 58.4% for the baseline model versus 78.5% and 70.3% for the enhanced model. ROC curves are depicted in Figure 3, illustrating the superior performance of the enhanced model.

**TABLE 6:** ROC analysis of predictive models for SALT50 achievement at week 24.

Model	AUC (95% CI)	Optimal cut-off	Sensitivity (%)	Specificity (%)	Youden index
Baseline model	0.69 (0.64–0.74)	0.46	71.2	58.4	0.296
Enhanced model	0.81 (0.76–0.85)	0.52	78.5	70.3	0.488

The baseline model included demographic and clinical covariates (age, sex, BMI, comorbidities). The enhanced model additionally incorporated disease activity status and disease duration. Optimal cut-off values were determined according to the maximum Youden index. AUC values were compared using the DeLong test. Sensitivity and specificity are reported at the optimal cut-off. SALT50, ≥50% improvement in SALT score from baseline.

### Relapse rate and time-to-relapse

**FIGURE 4:**
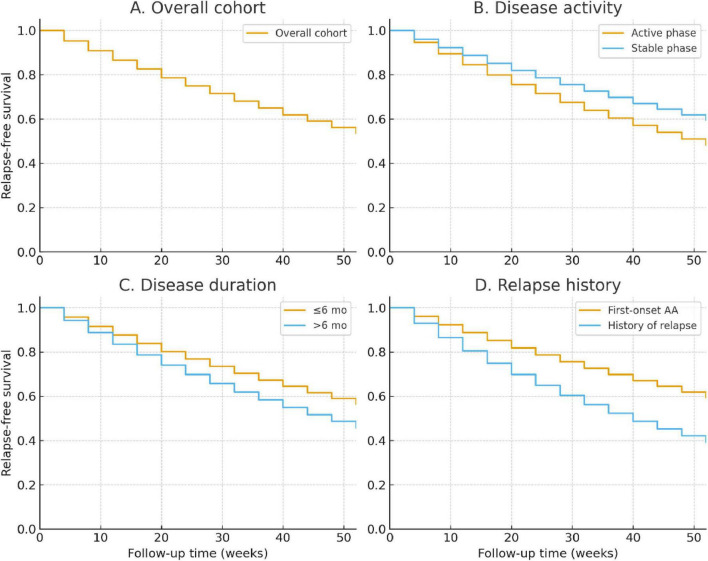
Kaplan–Meier analysis of relapse-free survival over 52 weeks. **(A)** Overall cohort. **(B)** Stratified by disease activity (active vs. stable phase). **(C)** Stratified by disease duration (≤6 months vs. >6 months). **(D)** Stratified by history of relapse (first-onset vs. relapsed cases). Log-rank tests showed significantly higher relapse risk in patients with longer disease duration and those with a prior history of relapse.

During the 52-week follow-up, 31.8% (127/400) of patients experienced relapse after achieving initial improvement. The median time to relapse was 28 weeks (IQR: 20–36 weeks). Kaplan–Meier analysis revealed a gradual decline in relapse-free survival, with an estimated 68.2% remaining relapse-free at week 52 (Figure 4).

Stratified analyses demonstrated significant differences across subgroups. Patients with disease duration >6 months had higher cumulative relapse risk than those with shorter duration (37.8% vs. 26.4%; log-rank *P* = 0.021; Figure 4). Similarly, patients in the active phase at baseline showed increased relapse compared with the stable phase (35.6% vs. 27.6%; log-rank *P* = 0.047; Figure 4). Those with a history of relapse were at the greatest risk, with nearly half relapsing by week 52 compared with 28.1% of first-onset cases (log-rank *P* = 0.009; Figure 4).

### Risk factors for relapse

Univariate Cox regression (Table 7) identified disease duration >6 months (HR 1.48, 95% CI 1.08–2.04, *P* = 0.015), active disease at baseline (HR 1.36, 95% CI 1.01–1.84, *P* = 0.042), and history of relapse (HR 1.67, 95% CI 1.22–2.29, *P* = 0.002) as significant predictors of relapse. Other variables, including age, sex, BMI, and comorbidities, were not associated with relapse risk. In the multivariate model, history of relapse remained the strongest independent predictor (HR 1.61, 95% CI 1.17–2.21, *P* = 0.004), while disease duration >6 months also conferred elevated risk (HR 1.44, 95% CI 1.04–2.00, *P* = 0.027). The effect of baseline disease activity was attenuated and no longer statistically significant (HR 1.28, 95% CI 0.95–1.74, *P* = 0.101). Exploratory subgroup analyses (Supplementary Table 1) indicated that the impact of disease duration on relapse was more pronounced among patients with stable disease, though the interaction test was non-significant (*P* = 0.742).

**TABLE 7:** Univariate and multivariate Cox regression analysis of predictors of relapse at 52 weeks.

Variable	Univariate HR (95% CI)	*P*-value	Multivariate HR (95% CI)	*P*-value
Age (per year)	1.01 (0.99–1.03)	0.242	1.00 (0.98–1.02)	0.761
Sex (female vs. male)	0.94 (0.68–1.29)	0.704	–	–
BMI (per kg/m^2^)	1.02 (0.97–1.06)	0.421	–	–
Disease duration >6 mo	1.48 (1.08–2.04)	0.015*	1.44 (1.04–2.00)	0.027*
Active disease	1.36 (1.01–1.84)	0.042*	1.28 (0.95–1.74)	0.101
History of relapse	1.67 (1.22–2.29)	0.002**	1.61 (1.17–2.21)	0.004**
Atopic dermatitis	1.21 (0.76–1.92)	0.416	1.15 (0.70–1.91)	0.573
Thyroid disorder	1.09 (0.55–2.16)	0.81	–	–
Anxiety/depression	1.17 (0.79–1.74)	0.431	–	–

*HR, hazard ratio; CI, confidence interval. Multivariate model adjusted for age, disease duration, disease activity, relapse history, and atopic dermatitis. *p < 0.05, *p < 0.01,

**p < 0.001.

### DLQI and Skindex-16 changes

Quality-of-life indices improved significantly throughout the 52-week follow-up. The mean DLQI score declined from 12.8 ± 6.1 at baseline to 8.4 ± 5.5 at week 12, 6.1 ± 4.9 at week 24, and 5.7 ± 4.6 at week 52. A similar trajectory was observed for the Skindex-16 total score, which decreased from 37.2 ± 12.5 at baseline to 23.5 ± 10.6 at week 24 and 21.8 ± 9.7 at week 52 (all *P* for trend < 0.001; Table 8).

**TABLE 8:** Changes in DLQI and Skindex-16 scores during 52-week follow-up.

Time point	DLQI (mean ± SD)	ΔDLQI from baseline	Skindex-16 total (mean ± SD)	ΔSkindex-16 from baseline	*P*-value (trend)
Baseline	12.8 ± 6.1	–	37.2 ± 12.5	–	–
Week 12	8.4 ± 5.5	–4.4 ± 3.6	28.6 ± 11.4	–8.6 ± 7.2	<0.001**
Week 24	6.1 ± 4.9	–6.7 ± 4.2	23.5 ± 10.6	–13.7 ± 8.1	<0.001**
Week 52	5.7 ± 4.6	–7.1 ± 4.5	21.8 ± 9.7	–15.4 ± 8.6	<0.001**

DLQI, Dermatology Life Quality Index; Skindex-16, 16-item Skindex dermatology quality-of-life scale. Δ values indicate mean change from baseline. *P*-values for trend derived from repeated-measures ANOVA.

**p < 0.001.

At week 24, 61.5% of patients achieved a clinically meaningful DLQI improvement (≥5-point reduction). This proportion was significantly higher among patients attaining SALT50 compared with non-responders (73.2% vs. 42.6%, *P* < 0.001), indicating that hair regrowth was closely linked to dermatology-specific quality-of-life improvement.

### Itch and pain symptom trajectories

**FIGURE 5:**
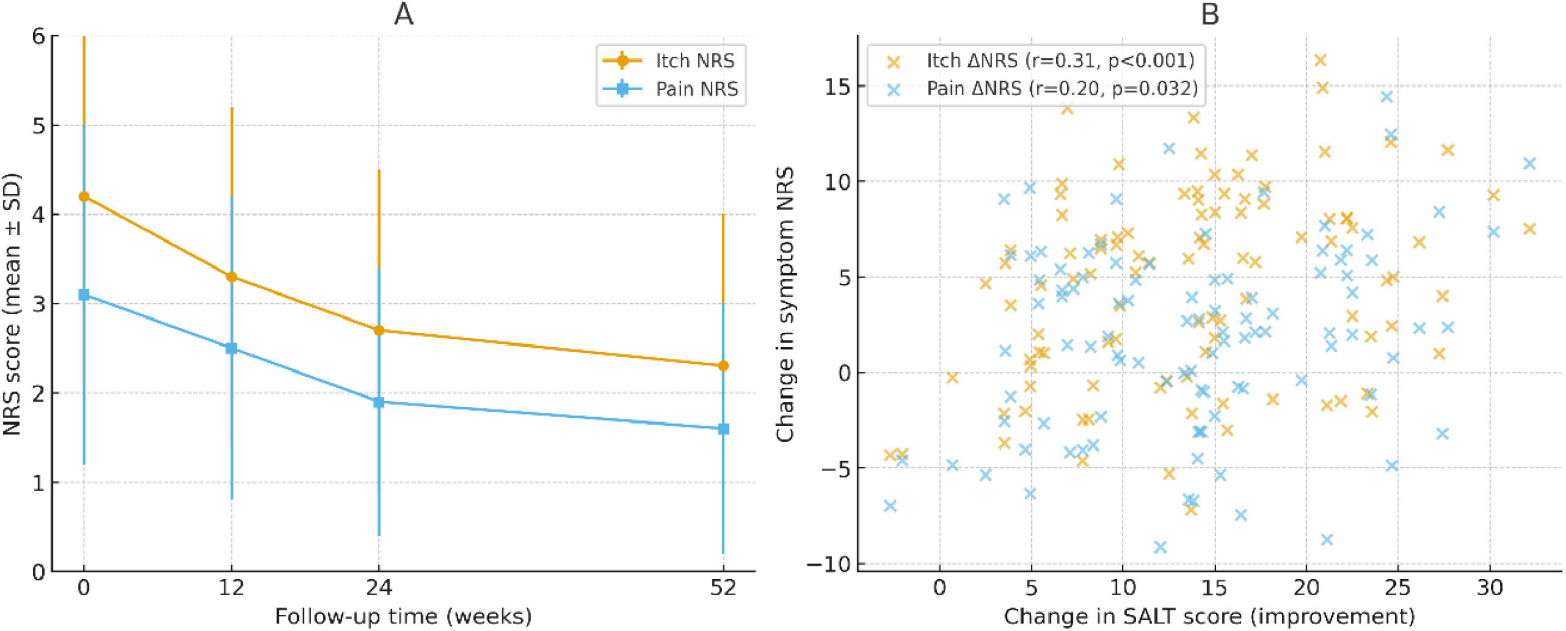
Changes in symptom scores and their correlation with hair regrowth. **(A)** Longitudinal trajectories of itch and pain numeric rating scale (NRS) scores over 52 weeks. Both symptoms showed progressive improvement, with the largest reductions observed by week 24 and sustained through week 52. **(B)** Scatter plots showing the correlation between changes in symptom NRS and improvements in SALT scores at week 24. Itch reduction correlated moderately with SALT improvement (Spearman *r* = 0.41, *P* < 0.001), whereas pain reduction showed a weaker but significant correlation (*r* = 0.23, *P* = 0.032). Trend lines are shown for visualization; correlations were tested using Spearman rank analysis.

Symptom burden also decreased over time. The mean itch NRS decreased from 4.2 ± 2.1 at baseline to 2.7 ± 1.8 at week 24 and 2.3 ± 1.7 at week 52. The pain NRS similarly improved from 3.1 ± 1.9 to 1.9 ± 1.5 at week 24 and 1.6 ± 1.4 at week 52, reflecting sustained symptomatic relief (Figure 5A). Correlation analyses showed that symptom improvement paralleled hair regrowth. At week 24, itch reduction was moderately correlated with SALT improvement (Spearman *r* = 0.41, *P* < 0.001), while pain reduction showed a weaker but significant correlation (*r* = 0.23, *P* = 0.032; Figure 5B).

### Baseline imaging parameters and disease severity

**FIGURE 6:**
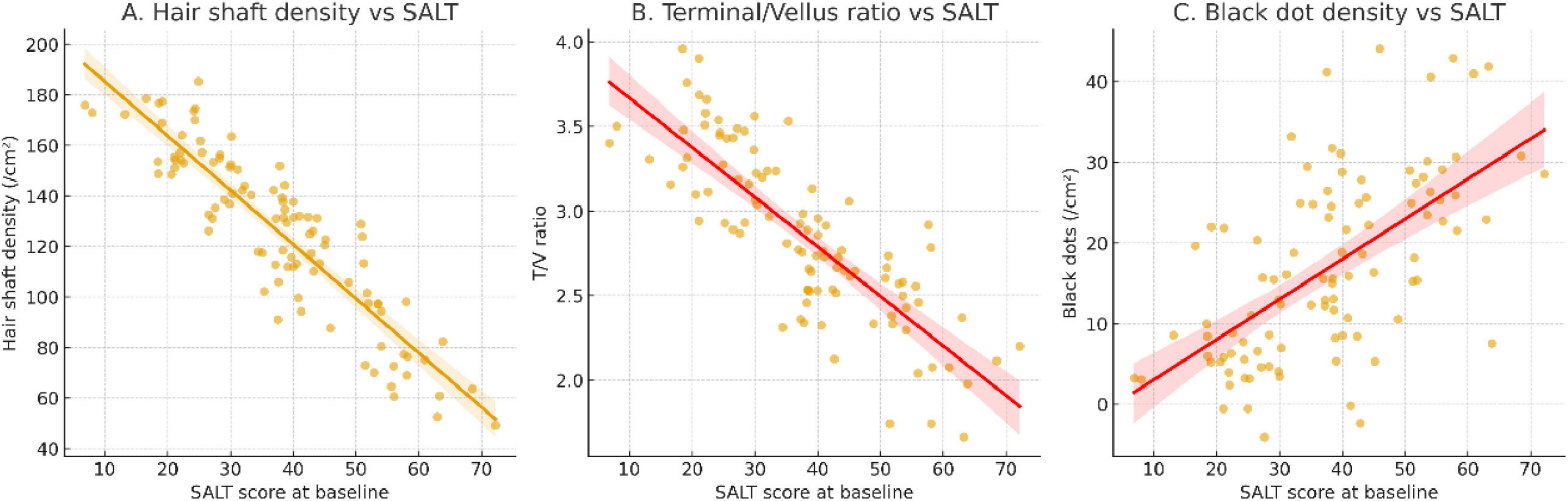
Baseline dermoscopic parameters and their correlation with SALT severity. **(A)** Hair shaft density showed a significant negative correlation with SALT score, indicating lower follicular density in patients with more severe alopecia. **(B)** Terminal-to-vellus hair ratio was inversely associated with SALT, reflecting miniaturization of follicles in severe disease. **(C)** Black dot density demonstrated a positive correlation with SALT score, highlighting its role as a dermoscopic marker of active disease. Regression lines are fitted for visualization; correlations were assessed using Spearman analysis.

In the imaging subcohort (*n* = 120), dermoscopic quantitative parameters were significantly associated with baseline disease severity. Hair shaft density (*r* = –0.46, *P* < 0.001), terminal-to-vellus hair ratio (*r* = –0.39, *P* = 0.002), and black dot density (*r* = 0.42, *P* < 0.001) all correlated with SALT scores, indicating that dermoscopic markers reliably reflected baseline alopecia burden (Figure 6). High-frequency ultrasound analyses revealed distinct differences between patients in the active and stable phases of alopecia areata. Dermal echogenicity heterogeneity was significantly higher in active disease (0.72 ± 0.14 vs. 0.61 ± 0.12, *P* = 0.004), reflecting greater tissue irregularity. Similarly, perifollicular hypoechoic banding was more prominent in active patients (65.3% ± 18.4% vs. 48.7% ± 16.9%, *P* < 0.001), and vascular signal intensity was elevated (1.82 ± 0.41 vs. 1.35 ± 0.37 a.u., *P* < 0.001). By contrast, follicular depth did not differ significantly between the two groups (2.17 ± 0.45 vs. 2.09 ± 0.42 mm, *P* = 0.428) (Table 9).

**TABLE 9:** Comparison of high-frequency ultrasound texture parameters between active and stable disease.

Parameter	Active phase (*n* = 60)	Stable phase (*n* = 60)	*P*-value
Dermal echogenicity heterogeneity	0.72 ± 0.14	0.61 ± 0.12	0.004**
Perifollicular hypoechoic banding (%)	65.3 ± 18.4	48.7 ± 16.9	<0.001**
Vascular signal intensity (a.u.)	1.82 ± 0.41	1.35 ± 0.37	<0.001**
Follicular depth (mm)	2.17 ± 0.45	2.09 ± 0.42	0.428

Data are presented as mean ± SD. a.u., arbitrary units.

**p < 0.001.

### Dynamic changes and prediction of treatment response

At week 12, early improvements in dermoscopic features were associated with subsequent treatment outcomes. Patients with ≥15% increase in hair shaft density or ≥20% reduction in black dot density were more likely to achieve SALT50 at week 24. ROC analysis demonstrated that the combined dermoscopic change index reached an AUC of 0.79 (95% CI: 0.71–0.86), outperforming individual parameters (AUC ∼0.69–0.70). When integrated with the baseline clinical model, imaging-derived changes further improved predictive accuracy. The imaging + clinical model achieved an AUC of 0.84 (95% CI: 0.77–0.89) compared with 0.76 (95% CI: 0.68–0.82) for the clinical model alone, with significant net reclassification improvement (NRI = 0.21) and integrated discrimination improvement (IDI = 0.07; *P* = 0.012) (Table 10).

**TABLE 10:** Predictive performance of imaging changes at week 12 for achieving SALT50 at week 24.

Model/ parameter	AUC (95% CI)	NRI	IDI	*P*-value vs. clinical model
Clinical model only (baseline covariates)	0.76 (0.68–0.82)	–	–	Reference
Hair shaft density Δ ≥ +15%	0.70 (0.63–0.77)	0.14	0.05	0.042*
Black dot density Δ ≤ −20%	0.69 (0.62–0.76)	0.12	0.04	0.051
Combined dermoscopic change index	0.79 (0.71–0.86)	0.18	0.06	0.018*
Imaging + clinical integrated model	0.84 (0.77–0.89)	0.21	0.07	0.012*

*AUC, area under the curve; NRI, net reclassification improvement; IDI, integrated discrimination improvement. *p* < 0.05.

### Safety and adverse events

Treatment was generally well tolerated over the 52-week follow-up. The overall incidence of adverse events (AEs) was 18.0%, with the majority classified as mild to moderate. The most frequent local corticosteroid–related reactions were skin atrophy and telangiectasia, while patients receiving systemic JAK inhibitors most commonly reported upper respiratory tract infections, transient liver enzyme elevations, and occasional neutropenia. Importantly, no severe opportunistic infections, thromboembolic events, or treatment-related deaths were observed. Detailed distributions of AE types, severity grading, and subgroup stratifications (by treatment modality, age, and sex) are provided in the Supplementary Table 2.

## Discussion

In this 52-week prospective cohort study, we comprehensively evaluated the clinical course, treatment outcomes, and patient-reported impacts of AA. Baseline SALT scores were higher in patients with active disease and prior relapse, while disease duration was less informative. Over time, SALT scores generally improved, though with notable heterogeneity across subgroups. At week 24, a substantial proportion of patients achieved SALT50 and SALT90, with baseline severity and activity serving as key predictors of response. By week 52, relapse was frequent, and Cox models identified baseline risk factors for recurrence. Quality-of-life scores (DLQI, Skindex-16) and symptom burden (itch and pain NRS) improved in parallel with clinical outcomes, highlighting the multidimensional impact of AA. Safety findings were consistent with known profiles of corticosteroids and JAK inhibitors, with no unexpected signals observed.

Our findings add to and extend the current understanding of alopecia areata. With respect to disease course and baseline severity, most prior studies have been retrospective or based on small sample sizes with limited follow-up (32–35). In contrast, our prospective design with 52 weeks of observation provides more robust longitudinal evidence. Consistent with earlier reports, we found that active disease status and relapse history were significantly associated with higher baseline SALT scores, whereas disease duration alone was less informative, underscoring the importance of disease activity over chronology in assessing severity (30, 37–43). Regarding therapeutic outcomes and predictors, our results support the growing body of evidence for the effectiveness of JAK inhibitors in real-world practice (44–49). Importantly, our study highlights baseline severity and activity status as major predictors of response, suggesting that patient stratification at treatment initiation may optimize outcomes.

The relapse rates observed over 52 weeks further emphasize the recurrent nature of AA. Differences in relapse proportions across studies may reflect variations in study design, treatment strategies, and patient demographics, as well as differences in the definition of relapse. In terms of quality of life and symptom burden, our results corroborate earlier findings that AA imposes a substantial psychosocial impact. However, by systematically incorporating DLQI and Skindex-16 alongside numeric symptom scales, our study provides one of the first longitudinal datasets to capture the parallel improvement in clinical severity and patient-reported outcomes. This dual perspective reinforces the need to integrate patient-centered measures into AA research and management. In addition, the inclusion of imaging parameters in a sub-cohort adds a novel dimension. The correlations between dermoscopic or ultrasound features and SALT scores support their potential role as objective biomarkers for disease monitoring and therapeutic prediction (38). Although preliminary, these findings suggest that imaging could complement clinical scoring systems in future precision management approaches.

Several mechanistic considerations may explain the clinical patterns observed in this study. From an immunological perspective, the higher SALT scores seen in patients with active disease likely reflect enhanced inflammatory activity around the hair follicle (37, 39). Previous studies have demonstrated infiltration of cytotoxic CD8^+^ NKG2D^+^ T cells and activation of Th1/Th17 pathways, with elevated cytokines such as IFN-γ, IL-15, and IL-17 driving follicular destruction (50–52). The recurrent nature of AA may be attributed to the repeated collapse of hair follicle immune privilege and the persistence of autoreactive memory T cells, which predispose patients to relapse even after apparent remission (53, 54). In terms of treatment response and relapse, the observed effectiveness of JAK inhibitors is consistent with their ability to block IFN-γ–driven JAK–STAT signaling, thereby disrupting key pathogenic pathways in AA (44, 46, 48, 52). However, the high rate of relapse after treatment discontinuation suggests that while JAK inhibition can suppress immune activity, it may not fully eliminate autoreactive immune memory, leaving the disease prone to recurrence once the inhibitory signal is removed (55, 56). Beyond immune modulation, the improvement in quality of life and symptom burden provides additional mechanistic insight. The positive correlation between reductions in SALT scores and decreases in itch and pain NRS suggests that resolution of perifollicular inflammation not only promotes hair regrowth but also directly alleviates sensory symptoms (39, 46, 57). This finding underscores the close link between inflammatory activity, clinical severity, and patient-reported outcomes in AA (37, 38).

The present findings have several important clinical implications. First, the identification of baseline SALT scores, disease activity, and relapse history as predictors of severity and outcomes may facilitate risk stratification and individualized treatment planning in patients with AA. Second, the study underscores the value of incorporating patient-reported outcomes, such as quality of life and symptom burden, as essential endpoints in clinical management. These measures not only reflect the broader impact of disease but also align therapeutic goals with patient priorities. From a research perspective, this prospective cohort provides valuable baseline data for future interventional studies and clinical trials of emerging therapies, including JAK inhibitors and other targeted agents.

This study has several notable strengths. The prospective design, large sample size, and 52-week follow-up allowed for systematic characterization of the natural course and treatment outcomes of AA, providing robust longitudinal evidence. The use of a multidimensional assessment framework, incorporating clinical severity, therapeutic response, relapse, quality of life, and imaging parameters, offers a more comprehensive understanding of disease burden than prior studies. Furthermore, the real-world design, which included patients receiving standard therapeutic pathways, enhances the generalizability and clinical relevance of the findings. Several limitations should also be acknowledged. First, the single-center setting may introduce selection bias, limiting the generalizability of the results. Second, although the 52-week follow-up is relatively long compared with previous studies, it remains insufficient to fully capture the long-term course and relapse patterns of AA beyond 2 years. Third, patients in this real-world cohort received heterogeneous treatment regimens, which may act as a potential confounder and complicate interpretation of treatment responses. The sample size of certain subgroups, particularly patients treated with JAK inhibitors, was limited, which may have reduced statistical power for subgroup analyses. Fourth, the imaging sub-cohort included only a subset of patients, thereby restricting the external validity of imaging-related findings. Finally, the absence of systematic molecular or immunological profiling means that mechanistic inferences remain indirect. Future research should aim to validate these findings in larger, multicenter cohorts, thereby enhancing external validity and generalizability. Longer follow-up periods, extending to at least 2 years or more, are needed to capture long-term remission and relapse patterns. Integrating molecular, immunological, and imaging biomarkers into prospective cohorts will be critical for advancing mechanistic understanding, improving prediction models, and guiding precision therapies in AA.

## Conclusion

In summary, this prospective 52-week cohort study provides a comprehensive characterization of the clinical course, therapeutic outcomes, and patient-reported impacts of alopecia areata. We demonstrated that baseline severity, disease activity, and relapse history are key determinants of treatment response and recurrence risk. Clinically meaningful improvements in SALT scores were paralleled by significant gains in quality of life and symptom relief, highlighting the multidimensional burden of the disease. The study also confirmed the safety profiles of conventional corticosteroid therapies and JAK inhibitors in a real-world setting. Together, these findings support the integration of standardized severity scoring, patient-centered outcomes, and imaging biomarkers into both clinical practice and future research, thereby laying the groundwork for risk stratification and personalized management strategies in AA.

## Data Availability

The original contributions presented in this study are included in this article/Supplementary material, further inquiries can be directed to the corresponding authors.
