# Supplementary material for: Longitudinal clinical course, treatment outcomes, and relapse patterns in alopecia areata: a prospective cohort study
Source: Front Med (Lausanne). 2025 Nov 14;12:1695618. doi: 10.3389/fmed.2025.1695618 (PMC12660087; doi:10.3389/fmed.2025.1695618)
Supplement: Supplementary file 1 [file Table_1.docx]

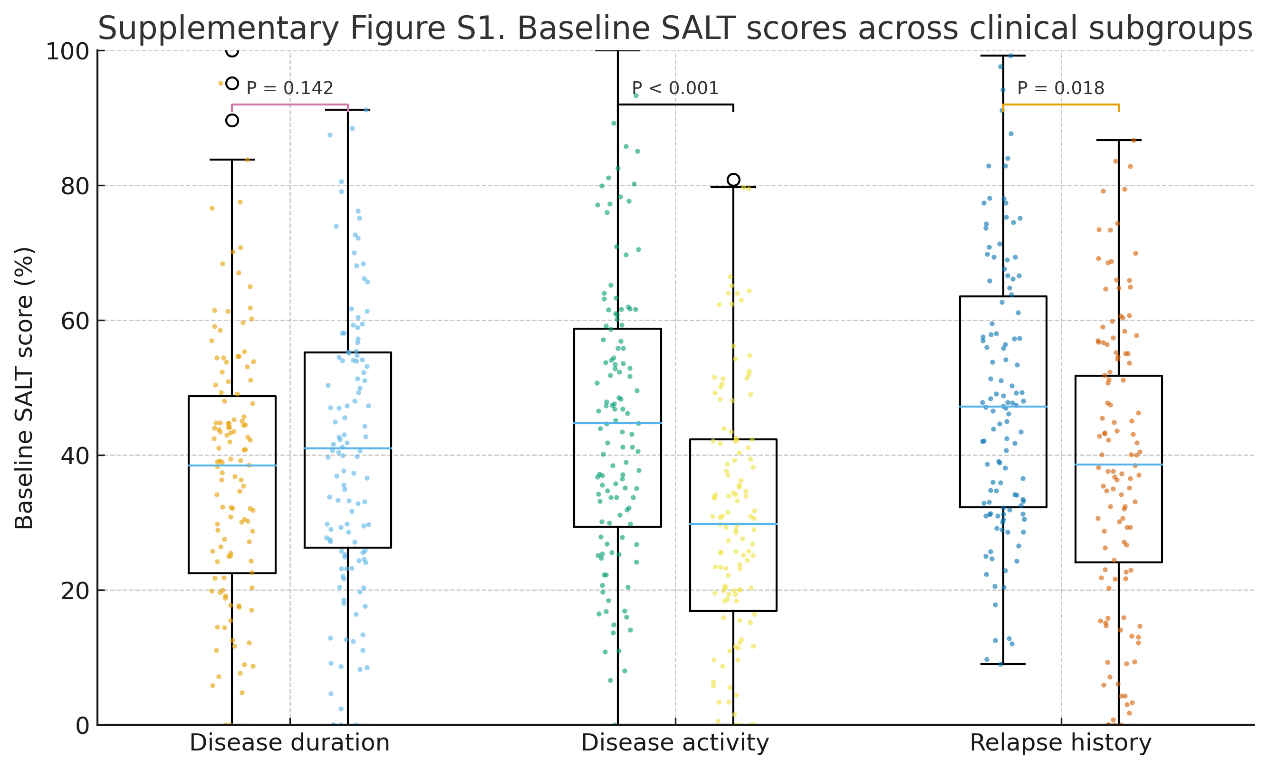


*Supplementary Figure S1. Baseline SALT scores according to disease duration, disease activity, and history of relapse. Box-and-whisker plots display the median, interquartile range, and range; overlaid jittered points show individual values. Patients in the active phase exhibited higher SALT scores than those in the stable phase (P < 0.001). Patients with a history of relapse also had higher scores than those without relapse (P = 0.018). No significant difference was observed between disease duration ≤ 6 months and > 6 months (P = 0.142).*

**Supplementary Table S1. Subgroup Cox regression analysis of relapse at 52 weeks**

| **Subgroup** | **HR for relapse (95% CI)** | **P value** |
| --- | --- | --- |
| **Disease activity** |  |  |
| Active phase (ref: Stable) | 1.28 (0.91–1.80) | 0.155 |
| **Disease duration** |  |  |
| ≤ 6 months (ref: >6 months) | 0.72 (0.51–1.02) | 0.061 |
| **Relapse history** |  |  |
| Prior relapse (ref: First-onset) | 1.58 (1.11–2.25) | 0.011 * |
| **Interaction terms** |  |  |
| Duration × Activity | 1.07 (0.71–1.61) | 0.742 |
| Relapse history × Activity | 1.12 (0.75–1.69) | 0.585 |

*HR = hazard ratio; CI = confidence interval. Subgroup Cox analyses adjusted for age and sex. Interaction terms tested whether the effect of duration or relapse history on relapse differed by disease activity. *p < 0.05.*

**Supplementary Table S2. Incidence and stratification of adverse events during 52-week follow-up**

| **Adverse event category** | **n (%)** | **Severity (mild/moderate/severe)** | **Stratified notes*** |
| --- | --- | --- | --- |
| **Local corticosteroid-related** |  |  |  |
| Skin atrophy | 20 (5.0) | 18 / 2 / 0 | More frequent in ≥40 years, females |
| Telangiectasia | 14 (3.5) | 14 / 0 / 0 | Even distribution across subgroups |
| **Systemic JAK inhibitor-related** |  |  |  |
| Upper respiratory infection | 10 (2.5) | 10 / 0 / 0 | Most common in JAKi users (9.1%) |
| Elevated liver enzymes | 6 (1.5) | 6 / 0 / 0 | More frequent in males |
| Neutropenia | 4 (1.0) | 3 / 1 / 0 | Observed only in JAKi users |
| **Other events** |  |  |  |
| Headache | 8 (2.0) | 8 / 0 / 0 | Slightly more common in <40 years |
| Injection site reaction | 10 (2.5) | 10 / 0 / 0 | Associated with local steroid injections |
| **Total adverse events** | 72 (18.0) | 69 / 3 / 0 | No treatment-related deaths |

*Stratified observations are based on subgroup analyses (age, sex, treatment modality). Detailed stratification percentages are available upon request.
